# Efficacy of immune checkpoint inhibitors in patients with KRAS-mutant advanced non-small cell lung cancer: A retrospective analysis

**DOI:** 10.1515/med-2023-0653

**Published:** 2023-03-10

**Authors:** Xiaodong Gu, Jinfei Si, Yelan Guan, Yibing Xu, Lan Shao, Yiping Zhang, Chunwei Xu, Weiwei Pan, Yuanzhi Lu, Zhengbo Song, Wenxian Wang

**Affiliations:** The Second Clinical Medical College of Zhejiang Chinese Medical University, Hangzhou, Zhejiang 310053, China; Department of Thoracic Medical Oncology, Chinese Academy of Sciences University Cancer Hospital (Zhejiang Cancer Hospital), Hangzhou, Zhejiang 310022, China; Department of Clinical Trial, Cancer Hospital of the University of Chinese Academy of Sciences (Zhejiang Cancer Hospital), Hangzhou, Zhejiang 310002, China; Department of Respiratory Medicine, Jinling Hospital, Nanjing University School of Medicine, Nanjing, Jiangsu 210002, China; Department of Cell Biology, College of Medicine, Jiaxing University, Jiaxing, Zhejiang 314001, China; Department of Clinical Pathology, The First Affiliated Hospital of Jinan University, Guangzhou, Guangdong 510630, China; Department of Clinical Trial, Cancer Hospital of the University of Chinese Academy of Sciences (Zhejiang Cancer Hospital), No. 1 Banshan East Street, Gongshu District, Hangzhou, Zhejiang 310002, China; Department of Thoracic Medical Oncology, Chinese Academy of Sciences University Cancer Hospital (Zhejiang Cancer Hospital), No. 1 Banshan East Street, Gongshu District, Hangzhou, Zhejiang 310002, China

**Keywords:** KRAS, NSCLC, ICIs, chemotherapy, KRAS G12C

## Abstract

The efficacy of immune checkpoint inhibitors (ICIs) on KRAS-mutant advanced non-small cell lung cancer (NSCLC) remains controversial. This retrospective study compared the effects of ICIs treatment and chemotherapy on the prognosis of patients with KRAS-mutant advanced NSCLC and different mutant subtypes in the real world. The study included 95 patients with KRAS-mutant advanced NSCLC. Patients treated with first-line ICIs plus platinum-containing chemotherapy had better progression-free survival (PFS) (7.4 vs 4.5 months, *P* = 0.035) and overall survival (OS) (24.1 vs 13.2 months, *P* = 0.007) than those receiving platinum-containing chemotherapy alone, and second-line ICI monotherapy was associated with better PFS (4.8 vs 3.0 months, *P* = 0.043) and OS (18.0 vs 13.8 months, *P* = 0.013) than chemotherapy monotherapy. There was no significant difference in PFS (5.267 vs 6.734 months, *P* = 0.969) and OS (19.933 vs 20.933 months, *P* = 0.808) between patients with KRAS-mutant and KRAS-wild-type NSCLC treated with ICIs or between KRAS G12C and KRAS non-G12C patients (PFS: 8.1 vs 4.8 months, *P* = 0.307; OS: 21.3 vs 21.8 months, *P* = 0.434). In summary, patients with advanced NSCLC with KRAS mutations can benefit from ICIs, but no difference between KRAS mutant subtypes was observed.

## 1 Introduction

Lung cancer incidence and mortality rates are among the highest of all malignancies worldwide [1], and non-small cell lung cancer (NSCLC) accounts for 85% of all lung cancers. Kirsten rat sarcoma viral oncogene homolog (KRAS) is one of the most commonly mutated oncogenes in NSCLC, with a mutation incidence of approximately 25% in Western populations [2]; however, the incidence of KRAS mutation in adenocarcinoma in the Asian population is 5–15% [3]. For a long time, the standard treatment for KRAS-mutant NSCLC patients has been cytotoxic chemotherapy. The emergence of tyrosine kinase inhibitors targeting epithelial growth factor receptor (EGFR) mutations marked the beginning of the era of precision medicine [4]. The KRAS protein lacks a suitable “pocket” for small-molecule binding, making it difficult to develop effective drugs against KRAS [5]. The inhibitors AMG 510 and MRTX849 targeting the KRAS G12C mutation have shown promise in early clinical trials [6,7]. However, the use of KRAS G12C inhibitors in the treatment of NSCLC is limited in the real world.

The development of immune checkpoint inhibitors (ICIs) profoundly changed the management of lung cancer, and in the past 5 years, ICIs have become the standard treatment for advanced NSCLC [8]. However, the efficacy of ICIs for NSCLC with known carcinogenic drivers is controversial [9–11]. The limited data on the efficacy of ICIs in patients with KRAS-mutant NSCLC are derived from subgroup analyses of large clinical studies. In addition, the effect of ICIs on KRAS mutant subtypes is rarely reported. Here, we designed a retrospective study to explore the efficacy of ICIs and the prognosis of patients with advanced NSCLC with KRAS mutation treated with ICIs in the real world. We also analyzed the differences in the efficacy of ICIs among KRAS mutant subtypes.

## 2 Methods

### 2.1 Study design

Patients with advanced KRAS-mutated NSCLC were grouped according to the number of lines of treatment and regimen. The first-line treatment was ICIs plus platinum-containing chemotherapy or platinum-containing chemotherapy alone, and the second-line treatment was ICI monotherapy or chemotherapy alone. All chemotherapy and ICI regimens were administered according to the standard doses established by the National Comprehensive Cancer Network guidelines.

### 2.2 Study population and selection criteria

Patients who were diagnosed with NSCLC in Zhejiang Cancer Hospital between March 2015 and March 2021 were retrospectively analyzed. The diagnosis of KRAS-mutant NSCLC was confirmed by real-time PCR or next generation sequencing. The enrolled patients met the following selection criteria: (1) patients diagnosed as NSCLC by pathology, and the histological classification of NSCLC was based on the World Health Organization criteria (2015 version) [12], (2) presence of KRAS mutations, and (3) complete data of baseline clinicopathological characteristics including age at diagnosis, gender, smoking history, histology, Eastern Cooperative Oncology Group performance status (ECOG PS) score, intrathoracic metastasis status, liver metastasis status, bone metastasis status, brain metastasis status, and KRAS mutation subtypes. Exclusion criteria were as follows: (1) other malignant disease histories at the time of diagnosis (because of the difficulty in calculating recurrent events and because double cancers may increase the risk of recurrence), (2) positive for EGFR, ALK, MET, ROS1, HER2, BRAF, RET, and NTRK, and (3) ECOG PS score of 3–4. In addition, we collected 58 patients with KRAS-wild-type advanced NSCLC treated with ICIs in our hospital during the same period who were matched according to the basic characteristics of KRAS-mutant patients.


**Ethics approval:** This study was approved by the Chinese Academy of Sciences University Cancer Hospital (Zhejiang Cancer Hospital) Ethics Committee (IRB-2022-33) and individual consent for this retrospective analysis was waived.
**Consent:** As this was a retrospective study, patient consent was waived, and anonymity was ensured.

### 2.3 Data collection

The medical records of patients with KRAS-mutant and KRAS-wild-type advanced NSCLC were collected, and baseline clinicopathological characteristics, treatment, and follow-up were recorded. Patient follow-up information was obtained from the last clinical visits, follow-up registration records, and follow-up phone records. The deadline for follow-up was December 31, 2021.

### 2.4 Assessments

Response to treatment was assessed using the Response Evaluation Criteria in Solid Tumors (RECIST) v1.1. Prior to analysis, efficacy was examined by two oncologists, who evaluated the tumor response according to RECIST 1.1 via chest computed tomography and/or brain magnetic resonance imaging every 4–8 weeks. Objective response rate (ORR) was defined as the proportion of patients with complete response plus partial response (PR). Progression-free survival (PFS) was defined as systemic progression or death from the date of initial treatment, or the date and time of the last follow-up, whichever comes first triggers the date review of the event. Overall survival (OS) was defined as the time from the diagnosis of advanced NSCLC to death or the last follow-up.

## 3 Results

### 3.1 Patient characteristics

Between March 2015 and March 2021, 95 patients with KRAS-mutant NSCLC were collected and included in the analysis. Of the 95 patients, 58 (61.1%) received ICIs as first- and second-line therapy. Regarding first-line therapy, 33 patients received ICIs plus platinum-based chemotherapy, and 37 received platinum-based chemotherapy. For second-line therapy, 25 patients received ICI monotherapy and ten patients who received platinum-based chemotherapy as first-line therapy received chemotherapy monotherapy. Among the 95 patients, 28 (29.5%) were younger than 60 years, 73 (76.8%) were male and 22 (23.3%) were female, 66 (69.5%) were former or current smokers, 91 (95.8%) were adenocarcinomas, 88 (92.6%) had ECOG PS score of 0–1, 52 (54.7%) had intrathoracic metastasis, six (6.3%) had liver metastasis, 33 (34.7%) had bone metastasis, 21 (22.1%) had brain metastasis, 23 (24.2%) were KRAS G12C, 42 (44.2%) were KRAS non-G12C, and 30 (31.6%) had unknown mutations. The patient characteristics at baseline are detailed in Table 1. We matched 58 KRAS-wild-type patients treated with ICIs according to the baseline characteristics of the 58 KRAS-mutant patients. The baseline characteristics of the 58 KRAS-wild-type patients are presented in Table 2.

**Table 1:** Baseline KRAS-mutant patient characteristics

Characteristic	All (*N* = 95)	ICIs (*N* = 58)	Chemotherapy (*N* = 37)	*P*
Age				
<60	28 (29.5)	17 (29.3)	11 (29.7)	0.965
≥60	67 (70.5)	41 (70.7)	26 (70.3)	
Gender				
Male	73 (76.8)	46 (79.3)	27 (73.0)	0.475
Female	22 (23.3)	12 (20.7)	10 (27.0)	
Smoking				
Yes	66 (69.5)	42 (72.4)	24 (64.9)	0.436
No	29 (30.5)	16 (27.6)	13 (35.1)	
Histology				
Adenocarcinoma	91 (95.8)	55 (94.8)	36 (97.3)	1.000
Non-adenocarcinoma	4 (4.2)	3 (5.2)	1 (2.7)	
ECOG PS score				
0–1	88 (92.6)	53 (91.4)	35 (94.6)	0.817
2	7 (7.4)	5 (8.6)	2 (5.4)	
Intrathoracic metastasis				
Yes	52 (54.7)	34 (58.6)	18 (48.6)	0.341
No	43 (45.3)	24 (41.4)	19 (51.4)	
Liver metastasis				
Yes	6 (6.3)	2 (3.4)	4 (10.8)	0.204
No	89 (93.7)	56 (96.6)	33 (89.2)	
Bone metastasis				
Yes	33 (34.7)	22 (37.9)	11 (29.7)	0.413
No	62 (65.3)	36 (62.1)	26 (70.3)	
Brain metastases				
Yes	21 (22.1)	14 (24.1)	7 (18.9)	0.550
No	74 (77.9)	44 (75.9)	30 (81.1)	
KRAS mutation subtypes				
G12C	23 (24.2)	12 (20.7)	11 (29.7)	0.103
Non-G12C	42 (44.2)	23 (39.7)	19 (51.4)	
Unknown	30 (31.6)	23 (39.7)	7 (18.9)	
PD-L1, TPS				
<1	10 (10.5)	6 (10.3)	4 (10.8)	0.394
1–49	35 (36.8)	24 (41.4)	11 (29.7)	
≥50	16 (16.8)	11 (19.0)	5 (13.5)	
Unknown	34 (35.8)	17 (29.3)	17 (45.9)	

ECOG PS, Eastern Cooperative Oncology Group Performance Status; PD-L1, programmed death ligand 1; TPS, tumor proportion score.

**Table 2:** Baseline patient characteristics

Characteristic	All (*N* = 116)	KRAS-mutant (*N* = 58)	KRAS-wild (*N* = 58)	*P*
Age				
<60	35 (30.2)	17 (29.3)	18 (31.0)	0.840
≥60	81 (69.8)	41 (70.7)	40 (69.0)	
Gender				
Male	87 (75.0)	46 (79.3)	41 (70.7)	0.284
Female	29 (25.0)	12 (20.7)	17 (29.3)	
Smoking				
Yes	78 (67.2)	42 (72.4)	36 (62.1)	0.235
No	38 (32.8)	16 (27.6)	22 (37.9)	
ECOG PS score				
0–1	109 (94.0)	53 (91.4)	56 (96.6)	0.435
2	7 (6.0)	5 (8.6)	2 (3.4)	
Intrathoracic metastasis				
Yes	70 (60.3)	34 (58.6)	36 (62.1)	0.704
No	46 (39.7)	24 (41.4)	22 (37.9)	
Liver metastasis				
Yes	6 (5.2)	2 (3.4)	4 (6.9)	0.675
No	110 (94.8)	56 (96.6)	54 (93.1)	
Bone metastasis				
Yes	44 (37.9)	22 (37.9)	22 (37.9)	1.000
No	72 (62.1)	36 (62.1)	36 (62.1)	
Brain metastases				
Yes	27 (23.3)	14 (24.1)	13 (22.4)	0.826
No	89 (76.7)	44 (75.9)	45 (77.6)	
PD-L1, TPS				
<1	16 (13.8)	6 (10.3)	10 (17.2)	0.181
1–49	38 (32.8)	24 (41.4)	14 (24.1)	
≥50	21 (18.1)	11 (19.0)	10 (17.2)	
Unknown	41 (35.3)	17 (29.3)	24 (41.4)	

ECOG PS, Eastern Cooperative Oncology Group Performance Status; PD-L1, programmed death ligand 1; TPS, tumor proportion score.

### 3.2 Efficacy

**Figure 1:**
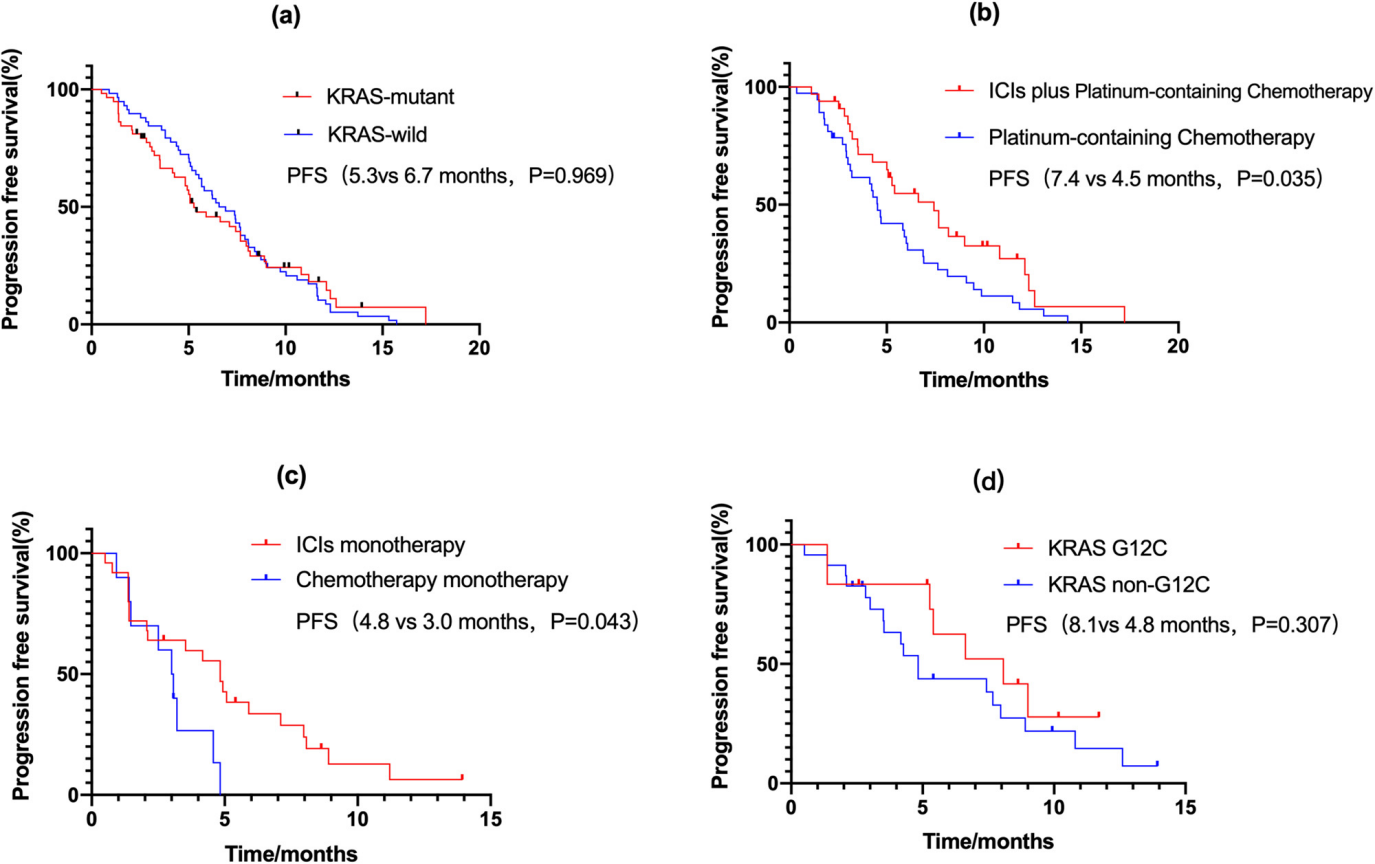
PFS (a) PFS of KRAS-mutant and KRAS-wild type; (b) PFS of first-line; (c) PFS of second-line; (d) PFS of KRAS-mutant subtypes. PFS: progression-free survival.

Of 58 KRAS-wild-type patients treated with ICIs, 16 (27.6%) had a PR and 36 (62.1%) were SD; the ORR and DCR were 27.6 and 89.7%, respectively, compared with 25.9 and 81.0% for the 58 KRAS-mutant patients treated with ICIs. No differences in mPFS (5.267 vs 6.734 months, *P* = 0.969, Figure 1a) were observed between KRAS-mutant and KRAS-wild-type patients.

There were 95 patients with KRAS-mutant NSCLC. Of 33 patients who received ICIs plus chemotherapy as the first-line treatment, nine (27.2%) achieved PR and 22 (66.7%) were in SD (ORR of 27.2% and DCR of 93.9%). The 37 patients who received chemotherapy as the first-line treatment had an ORR of 16.2% and DCR of 75.7%. Patients treated with ICIs plus chemotherapy had a better mPFS than those receiving chemotherapy alone (7.4 vs 4.5 months, *P* = 0.035, Figure 1b). For second-line therapy, the ICI monotherapy group had an ORR of 24% and DCR of 64%, whereas the chemotherapy monotherapy group had an ORR of 0% and DCR of 70%. Similarly, patients receiving ICI monotherapy had a better mPFS than those receiving chemotherapy monotherapy (4.8 vs 3.0 months, *P* = 0.043, Figure 1c).

**Figure 2:**
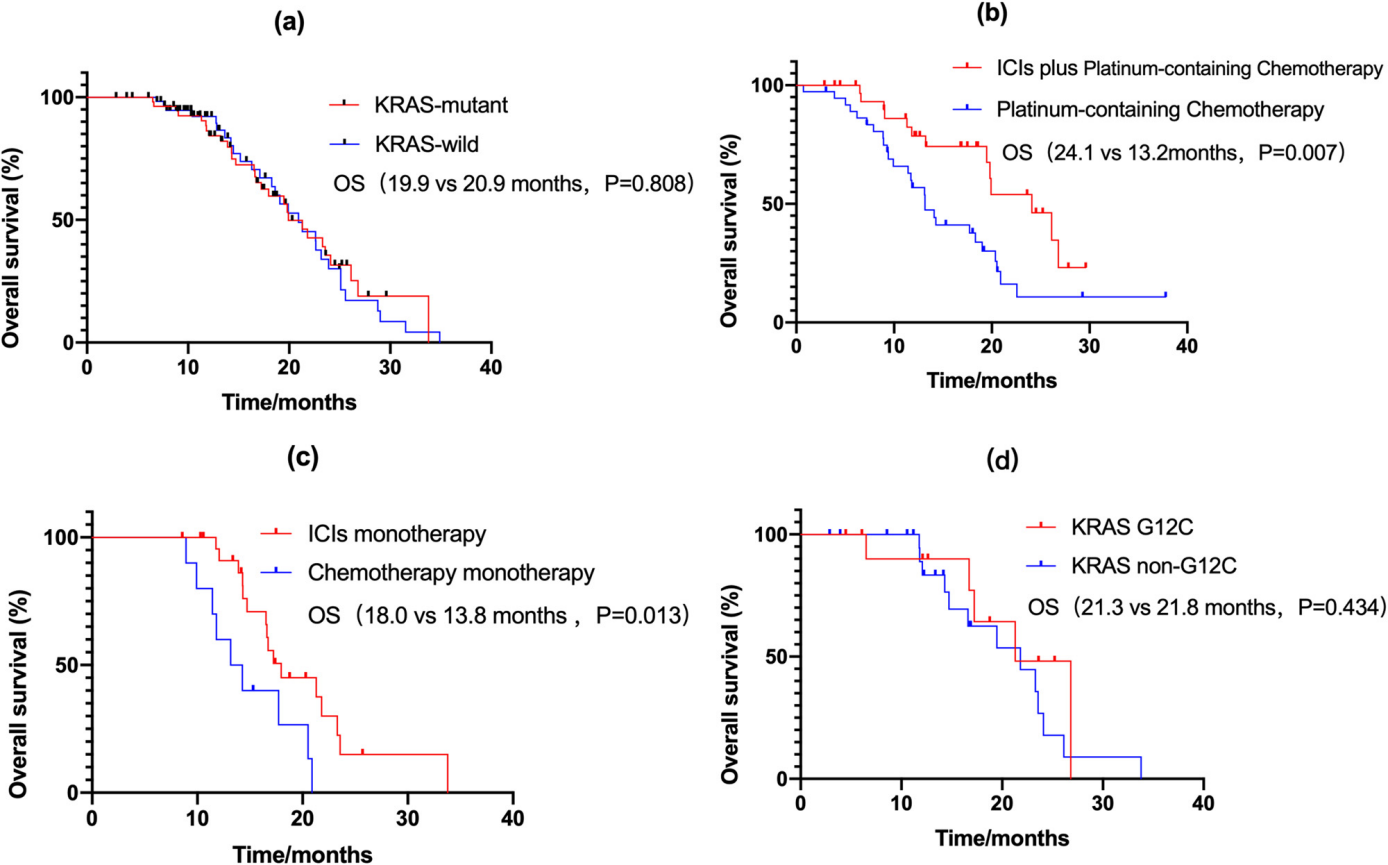
OS (a) OS of KRAS-mutant and KRAS-wild type; (b) OS of first-line; (c) OS of second-line; (d) OS of KRAS-mutant subtypes. OS: overall survival.

Among the 58 KRAS-mutant NSCLC patients who received ICIs, 35 had clear KRAS mutation subtypes. The distribution of subtypes was as follows: G12C in 12 cases (34%), G12D in nine cases (26%), G12V in nine cases (26%), G12R in one case (3%), G12S in two cases (6%), G12A in one case (3%), and G13C in one case (3%). The 35 patients were divided into KRAS G12C and KRAS non-G12C. Of 12 patients with KRAS G12C, three achieved PR, and of 23 patients with KRAS non-G12C, three achieved PR; the ORR was 25 vs 13% (*P* = 0.391). There were no significant differences in mPFS between patients with or without KRAS G12C (8.1 vs 4.8 months, *P* = 0.307, Figure 2d).

### 3.3 OS

At the cutoff date (March 31, 2021), the median follow-up time was 23.600 months (95% confidence interval [CI], 18.014–29.186). There were no significant differences in mOS between KRAS-mutant and KRAS-wild type patients (19.933 vs 20.933 months, *P* = 0.808, Figure 2a). The mOS was significantly better in patients receiving ICIs as the first-line (24.1 vs 13.2 months, *P* = 0.007, Figure 2b) or second-line (18.0 vs 13.8 months, *P* = 0.013, Figure 2c) treatment than in those receiving chemotherapy alone. There were no significant differences in mOS between patients with or without KRAS G12C (21.3 vs 21.8 months, *P* = 0.434, Figure 2d).

Univariate analysis included age (<60 vs ≥60), gender (male vs female), smoking status (current/former vs never), ECOG PS score (0–1 vs 2), intrathoracic metastasis (yes vs no), liver metastasis (yes vs no), bone metastasis (yes vs no), brain metastasis (yes vs no), ICIs treatment (yes vs no), programmed death ligand 1 (PD-L1) tumor proportion score (TPS) (≥1% vs <1%), and PD-L1 TPS (unknown vs <1%) (as variables). In the univariate analysis, ECOG PS score 0–1 (hazard ratio [HR] 0.314, *P* = 0.006), ICI treatment (HR 0.478, *P* = 0.006), and PD-L1 TPS ≥1% were associated with better OS. Multivariate analysis confirmed that ECOG PS score 0–1 (HR 0.336, *P* = 0.017), ICI treatment (HR 0.464, *P* = 0.007), and PD-L1 TPS ≥1% (HR 0.361, *P* = 0.023) were positive prognostic factors for OS, and liver metastasis (HR 2.869, *P* = 0.037) was a negative prognostic factor for OS (Table 3).

**Table 3:** Univariate and multivariate analyses of factors associated with OS

	Univariate		Multivariate	
	HR (95% CI)	*P*-value	HR (95% CI)	*P*-value
Age (<60 vs ≥60)	0.632 (0.344–1.161)	0.139	—	—
Gender (male vs female)	0.672 (0.358–1.261)	0.216	—	—
Smoking (current/former vs never)	1.074 (0.606–1.903)	0.806	—	—
ECOG PS score (0–1 vs 2)	0.314 (0.137–0.719)	0.006	0.336 (0.137–0.826)	0.017
Intrathoracic metastasis (yes vs no)	0.826 (0.489–1.398)	0.477	—	—
Liver metastasis (yes vs no)	2.268 (0.892–5.765)	0.085	2.869 (1.066–7.719)	0.037
Bone metastasis (yes vs no)	1.304 (0.765–2.221)	0.329	—	—
Brain metastases (yes vs no)	0.618 (0.302–1.265)	0.188	—	—
ICIs treatment (yes vs no)	0.478 (0.281–0.812)	0.006	0.464 (0.266–0.808)	0.007
PD-L1 TPS (≥1% vs <1%)	0.336 (0.150–0.751)	0.008	0.361 (0.150–0.869)	0.023
PD-L1 TPS (unknown vs <1%)	0.742 (0.346–1.591)	0.443	0.834 (0.376–1.852)	0.656

## 4 Discussion

This retrospective study explored the response to treatment and prognosis of patients with KRAS-mutant NSCLC who received ICIs with or without combination chemotherapy as first- or second-line treatments compared with chemotherapy alone, compared with published clinical studies of NSCLC patients who received ICIs. There are no published prospective clinical studies of patients with KRAS-mutated NSCLC who received ICIs, and the existing literature is limited to subgroup analyses or meta-analyses of large clinical studies. The findings of the present study show that PFS and OS were significantly prolonged by ICIs with or without chemotherapy compared with chemotherapy combination or monotherapy as first- and second-line treatments.

Several studies have evaluated the efficacy of ICIs in patients with advanced KRAS-mutant NSCLC, although the results are controversial. Retrospective studies and subgroup analyses of prospective studies showed that in patients with KRAS-mutant NSCLC, ICIs have better efficacy than chemotherapy [13–18]. Subgroup analysis in the CheckMate057 study showed that the OS benefit of nivolumab monotherapy as second-line treatment was better than that of docetaxel in NSCLC patients with KRAS mutations (HR = 0.52; 95% CI: 0.29–0.95), whereas the OS benefit was limited in patients with KRAS-wild-type NSCLC (HR = 0.98; 95% CI: 0.29–0.95) [16]. In addition, KEYNOTE-189 subgroup analysis showed that both PFS (HR = 0.79; 95% CI: 0.45–1.38) and OS (HR = 0.79; 95% CI: 0.45–1.38) were better with first-line pembrolizumab plus chemotherapy than with chemotherapy alone in patients with advanced KRAS mutations [17]. A meta-analysis concluded that for NSCLC with KRAS mutations, ICIs with or without chemotherapy significantly prolonged PFS (HR = 0.58 (0.43–0.78), *P* = 0.0003) and OS (HR = 0.59 (0.49–0.72), *P* < 0.00001) compared with chemotherapy monotherapy in both first- and second-line trials [18]. In contrast, some retrospective studies suggested that the efficacy of ICIs in patients with advanced NSCLC is independent from the KRAS mutation status [19,20]. The clinical efficacy of ICIs in KRAS mutant NSCLC remains a topic of debate. In this study, first-line ICIs plus chemotherapy led to better PFS (7.4 vs 4.5 months, *P* = 0.035) and OS (24.1 vs 13.2 months, *P* = 0.007) than chemotherapy. Second-line ICI monotherapy was also associated with better PFS (4.8 vs 3.0 months, *P* = 0.043) and OS (18.0 vs 13.8 months, *P* = 0.013) than chemotherapy monotherapy. In addition, we compared KRAS-mutant and KRAS-wild-type patients treated with ICIs and found no statistically significant difference in PFS (5.267 vs 6.734 months, *P* = 0.969) and OS (19.933 vs 20.933 months, *P* = 0.808). These results support that KRAS-mutant patients benefit from ICIs both as first- and second-line treatment, and the benefit is similar in patients with KRAS-wild-type NSCLC.

Given the heterogeneity of KRAS mutations, KRAS G12 status was divided into G12C and non-G12C. Studies suggest that KRAS G12 status is not related to the efficacy of ICIs [19–22]. However, in a retrospective analysis that included 168 KRAS G12C and 219 KRAS non-G12C patients with advanced NSCLC treated with ICIs, ORR, PFS, and OS were better in patients with KRAS G12C mutations. In the present study [23], the efficacy of ICIs did not differ significantly between KRAS G12C and non-G12C patients regarding PFS (8.1 vs 4.8 months, *P* = 0.307) and OS (21.3 vs 21.8 months, *P* = 0.434).

In addition to the KRAS mutation subtypes, co-mutation plays an important role in the efficacy of ICIs. Co-mutation of KEAP1/NFE2L2 with KRAS is associated with poorer ICIs efficacy [2]. Furthermore, the KRAS gene is associated with a high tumor mutational burden, CD8+ tumor cell infiltration, and high programmed death ligand 1 expression, which may be associated with better efficacy of ICIs [15].

The present study had several limitations. First, the retrospective nature of the study may have affected the results. Second, information on KRAS-mutant subtypes and co-mutation genes was not available for all patients. In addition, the choice of drugs for ICIs and chemotherapy was not uniform, and the related data were thus different. Finally, the sample size was relatively small; prospective or larger sample size studies are needed to validate the results.

## 5 Conclusions

Patients with advanced NSCLC with KRAS mutations can benefit from ICIs, although no difference between KRAS mutant subtypes was observed.

## Abbreviations

CI: confidence interval
ECOG PS: Eastern Cooperative Oncology Group Performance Status
EGFR: epithelial growth factor receptor
HR: hazard ratio
ICIs: immune checkpoint inhibitors
KRAS: Kirsten rat sarcoma viral oncogene homolog
NSCLC: non-small cell lung cancer
ORR: objective response rate
OS: overall survival
PD-L1: programmed death ligand 1
PFS: progression-free survival
PR: partial response
RECIST: Response Evaluation Criteria in Solid Tumors
TPS: tumor proportion score
